# Effect of Curdlan on the Rheological Properties of Hydroxypropyl Methylcellulose

**DOI:** 10.3390/foods10010034

**Published:** 2020-12-24

**Authors:** Liang Zhang, Li-Na Yue, Jian-Ya Qian, Xiang-Li Ding

**Affiliations:** 1 School of Food Science and Engineering, Yangzhou University, Huayang Xilu 196, Yangzhou 225127, China; zhangliang@yzu.edu.cn (L.Z.); ln2243641817@gmail.com (L.-N.Y.); dingxl@yzu.edu.cn (X.-L.D.); 2 Jiangsu Key Laboratory of Dairy Biotechnology and Safety Control, Yangzhou University, Huayang Xilu 196, Yangzhou 225127, China

**Keywords:** hydroxypropyl methylcellulose, curdlan, thermal gel, rheological property

## Abstract

This work focuses on the effect of curdlan (CL) on dynamic viscoelastic property, thermal reversible property, viscosity, and the fluid types of hydroxypropyl methylcellulose (HPMC) at different temperatures. Compared to the blends at 25 °C, the blends had a smaller linear viscoelastic region (LVR), a higher gel strength, and larger storage modulus (G’) and loss modulus (G”) values at 82 °C. G’, G”, gel strength, and viscosity increased with the increase of CL. Repeated temperature sweep led to increased G’ and G” of HPMC/CL blends. For HC6 and HC8, the gel formation temperature of the repeated temperature sweep was significantly lower than that of the first sweep. The samples at 82 °C, except for the sample with 8% CL, were all yield-shear thinning fluids, and the samples at 40 °C were shear thinning fluids. The creation of HPMC/CL and its rheological research might provide some methodological references for the study of other thermal–thermal gel blends.

## 1. Introduction

Hydroxypropyl methylcellulose (HPMC) is a modified cellulose derivative that can be widely used in the food industry as pre-coatings for fried foods, edible film for food preservation, thickening agent, suspension agent, and slow-release material for food and medical usage, etc., due to its thermal gel, film-forming properties, thickening property, dispersibility, and solubility [1,2,3,4]. HPMC can always display thermo-gel behavior; however, the gel formation is temperature- and concentration-dependent [5,6,7,8,9]. Studying the rheological properties of HPMC and their blends is of great significance for their application. Talukdar et al. studied the rheological properties of xanthan gum (XG) and HPMC by oscillatory and steady shear measurements at lower temperatures [10]. XG solution exhibits “gel-like” behavior in pure water and phosphate buffer at pH 7.4, while HPMC behaves as a typical polymer solution [10]. The addition of HPMC reinforced the gel properties and thermal stability of fish skin gelatin (FG) in this system, and the composite gel exhibited reversible cold and thermal gelation properties [11]. These characteristics can extend the scope of applications for FG in the food industry, particularly in edible casing applications for cold-storage and hot-service products [11]. There are also studies on the rheological properties of composite gel based on HPMC and other cold gels, such as hydroxypropyl starch (HPS) and collagen, which have enhanced the viscosity of HPMC at lower temperatures. Additionally, their blends may form gels at both lower and higher temperatures [12,13]. To conclude, most of the previous research has focused on the rheological properties of HPMC and cold gels. Research on the rheological properties and composite gel behavior of HPMC blends with thermal gels are deficient, which can lay the foundation of their application as hot-service products, such as synergetic thickening agent and pre-coating materials for fried foods.

Curdlan (CL), a linear triple helix polysaccharide, composed of one 3-β-linked D-glucose unit produced by a strain of *Alcaligenes faecalis* [14], is a hot gel [15]. Since it was first found by Harada in 1966, CL has received great attention in food and non-food industries due to its unique physicochemical properties [16]. It is insoluble in water, alcohol, or acid solution but soluble in alkaline solutions (pH ≥ 12), such as sodium hydroxide and trisodium phosphate. Although it is insoluble in water, CL is able to form two types of gels after heating: a low-set gel and a high-set gel. The low-set CL gel is a thermo-reversible gel that is formed when an aqueous dispersion is heated to a relatively low temperature between 55 and 65 °C [17]. However, when this aqueous dispersion is heated to a much higher temperature, it can form a thermo-irreversible gel, which is much more stable, not only at low temperatures (freezing) but also at high temperatures [17]. In low-set gel, cross-linking occurs between CL micelles, which are occupied by molecules of a single-helix through hydrogen bonds, whereas, in the high-set gel, the CL micelles are cross-linked by a triple-stranded helix through hydrophobic interactions [18]. Due to its unique thermal gelling properties, CL has been widely used in various foods, such as bean curd, noodles, jellies, and low-fat meat products [19].

So far, no document has been retrieved on the rheological and gel properties of HPMC/CL blends. This research intended to study the rheological property of thermal–thermal blending gels, including dynamic viscoelastic properties, viscosity, and fluid types at different temperatures, and thermal reversible properties. Furthermore, the morphology of the blends was studied, as well. The establishment of this thermal-thermal blending gel system and research on its rheological properties might provide some methodological and theoretical references for other thermal–thermal blending gels. This research might also provide some theoretical guidance for the application, such as a synergetic thickening agent or pre-coatings for fried foods of HPMC/CL blends.

## 2. Materials and Methods

### 2.1. Materials

HPMC (ZW-E6) (methoxyl content: 29%; hydroxypropyl oxygen content: 8.4%; the viscosity of 2% (*w*/*w*) HPMC: 6 mPa.s; weight-average molecular weight (Mw): 2.441 × 10^4^ g/mol) was bought from Hopetop Pharmaceutical Company (Huzhou, China). A food-grade CL (CG-01, 83% purity) was purchased from Jiangsu Yiming Biological Technology Co., Ltd. (Suqian, China).

### 2.2. Sample Preparation

HPMC was dispersed in deionized hot water (90 °C) by slowly agitating for 20 min, then it was cooled to room temperature by stirring at 30 rpm for 40 min to prepare 10% (*w*/*w*) HPMC, followed by the addition of CL to prepare the HPMC/CL system with 10% HPMC (*w*/*w*) and 0%, 2%, 4%, 6%, and 8% CL (*w*_CL_/*w*_(water+HPMC)_), respectively. Finally, the above suspensions were continuously stirred at 60 rpm for 1 h and at 40 rpm for another 1 h at room temperature before a rheological test. HC0, HC2, HC4, HC6, and HC8 were used to mark the five suspensions, respectively.

### 2.3. Rheological Behaviors

A strain sweep, frequency sweep, temperature sweep, and shear rate sweep were used to measure the rheological properties of this HPMC/CL blend system by using a controlled stress Rheometer (Kinexus Pro+, Malvern Instruments, Worcestershire, UK). The plate rotor PU40 SR1343 SS, with a diameter of 40 mm and a gap of 1.0 mm, was chosen for the strain sweep, frequency sweep, and temperature sweep, while the cylindrical rotor C25 SW1114 SS, with a diameter of 25 mm and a gap of 1.0 mm, was chosen for the shear rate sweep. Except for the temperature sweep, pretreating was done for all other sweeps. For pretreating, all samples were heated to 95 °C and maintained for 5 min in the sample holder; they were then cooled to 82 °C and 25 °C, respectively. The sweep began when the sample stabilized. All the samples tested at higher temperatures were sealed with silicone oil to prevent water evaporation. The specific methods are described below:

The strain sweep was mainly carried out to obtain the linear viscoelastic region (LVR) [20,21]. For the strain sweep, samples were tested in the range of 0.01–100%, with the tested frequencies of 1 Hz at 25 °C and 82 °C, respectively. The fixed amplitude of 0.1% was chosen for the frequency sweep and temperature sweep because, with this constant amplitude, the linear region was not surpassed in all cases, as is shown later.

The frequency sweep was tested in the range of 0.1–10 Hz, with the tested strains of 0.1% at 25 °C and 82 °C, respectively.

The shear rate sweep was tested using the procedure of the Toolkit_V001–1 Table of Shear Rates/Equilibrium Flow Curve in the range of 1–500/s at 40 °C and 82 °C, respectively. The flow pattern could be ascertained by fitting the shear stress (*σ*) and shear rate values (γ) into the corresponding fluid equations. The fluid equation corresponded to the shear thinning fluid and the yield-shear thinning fluid, as displayed below, respectively:

(1) $$\sigma =K\gamma^{n}$$

(2) $$\sigma =K\gamma^{n}+\sigma_{y}$$

where, the fluid consistency index (*K*), the flow behavior index (*n*), and yield stress (*σ_y_*) can be calculated.

The temperature sweep was tested in the range of 25–90 °C. A heating rate of 2 °C/min, a strain of 0.1%, and a frequency of 1 Hz were set in the experimental work. The samples were sealed with silicone oil to prevent water evaporation. Immediately after the first sweep, a repeated second temperature sweep was performed under the same conditions. The two temperature sweeps were differentiated as −1 and −2, for example, HC0-1 for the first time and HC0-2 for the second time. For HC8, another temperature sweep in the range of 25–95 °C was carried out twice as a comparison to prove that pretreating temperature greatly affected the thermal-irreversible gel formation. The second temperature sweeps of HC8, after the first temperature sweep heating to 90 °C and 95 °C, were recorded and written as H90-2 and H95-2, respectively.

All the rheological tests were tested three times, and the data used in the figures are the average data of the three tests.

### 2.4. Scanning Electron Microscopy (SEM)

Samples of HC0, HC2, and HC6 (prepared as Section 2.2) were placed in a water bath of 82 °C for 5 min to form gel. According to the method of Zhang, Lim, and Chung [22], with a few modifications, the gels were pre-frozen in liquid nitrogen for 3 min put in the refrigerator at −80 °C for 12 h, and lyophilized by using a freeze dryer (Alpha 1–2 LD Plus, Christ, Osterode, Germany). The lyophilized samples were stored in a desiccator before SEM measurement.

The lyophilized HPMC/CL blends were coated with gold-palladium and observed with environmental scanning electron microscopy (XL 30 ESEM, Philips, Amsterdam, The Netherlands) at 10 kV.

### 2.5. Statistical Analysis

SPSS Statistics 19 (IBM, Armonk, NY, USA) was used to analyze the data. The data were presented as the mean ± standard deviations (SDs). The mean was compared using one-way analysis of variance (ANOVA) followed by Duncan’s multiple comparison tests. Different superscript letters mean that values differed significantly (*p* < 0.05).

## 3. Results and Discussion

### 3.1. The Effect of CL on the Linear Viscoelastic Region of the HPMC System

**Figure 1:**
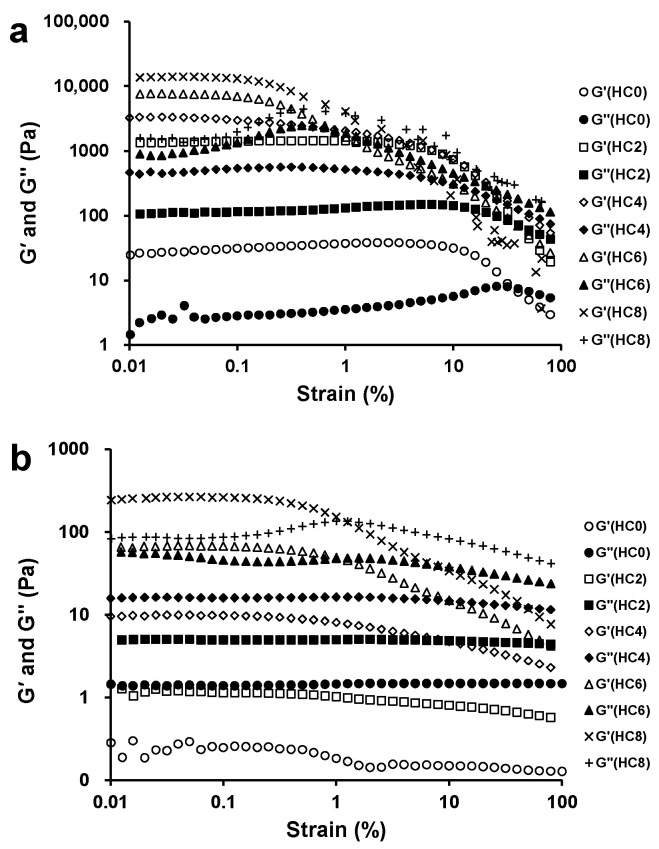
Strain sweep curves of hydroxypropyl methylcellulose/curdlan (HPMC/CL) blends at 82 °C (**a**) and 25 °C (**b**).

The typical curve of the strain sweep obtained for HPMC/CL blends at 82 °C is shown in Figure 1a. In these curves, G’, the storage modulus, describes the elasticity of polymers; whereas, G”, the loss modulus, reflects the viscous properties [23]. For HC0, when the strain was less than 7.47 ± 2.78%, G’ was stable; when the strain was more than 7.47 ± 2.78%, G’ had a large drop and entered the yielding zone, indicating that the LVR of HC0 was ≤ 7.47 ± 2.78%. G” began to rise and surpassed G’ when the strain was at 43.45 ± 5.97%, indicating that gel structure was damaged and the sample was at fluid state (G” > G’). For HC2, HC4, HC6, and HC8, the gel structures were damaged at 40.28 ± 0.10%, 40.11 ± 0.10%, 0.72 ± 0.31%, and 0.66 ± 0.49%, respectively. The LVRs of HC2, HC4, HC6, and HC8 were ≤2.57 ± 0.90%, ≤0.36 ± 0.26%, ≤0.15 ± 0.05%, and ≤0.20 ± 0.10%, respectively. The LVR of the sample became narrower after adding CL, in other words, the elasticity of the gels decreased. The G’ in the LVRs (written as the G’ of the critical point of the LVR region) were 25.8 ± 9.6 Pa, 1317.3 ± 142.7 Pa, 2514.7 ± 714.9 Pa, 7146.0 ± 712.1 Pa, and 12,833.3 ± 1020.3 Pa for samples HC0, HC2, HC4, HC6, and HC8, respectively. The G’ in the LVRs can be used to represent gel strength. Thus, the gel strength of the sample increased with the increased CL content, and the increasing extent of HC8 was the largest, at about 500 times higher than that of HC0. In conclusion, the gel strength increased with the CL content, while the LVR decreased, which may be because, with the increase of CL content, the blending gels were rigid and brittle.

The effect of CL on the strain sweep curve of HPMC/CL blends at 25 °C is presented in Figure 1b. For HC6 and HC8, G’ was larger than G” in the range of less than 1.13 ± 0.18% and 1.26 ± 0.00% at 25 °C, respectively, indicating that HC6 and HC8 were in the gel state at a lower strain range. Since HPMC can display thermo-reversible gel property, when it was heated to the gel formation temperature, the HPMC solution could turn into gel; when it was cooled to a certain temperature, the sample could change into the solution again. Meanwhile, CL could form thermo-irreversible gel when the processing temperature was high enough and the gel formation could not be disassembled again after cooling, since it turned thermo-irreversible. Here, HC6 and HC8 presents gel property at 25 °C, which may be because in these samples, CL formed thermo-irreversible gel after being pretreated at 95 °C. All the other samples with lower CL content showed G” > G’, indicating that these samples exhibited sol-like behavior. For samples with sol-like behavior, the viscous behavior was superior to the elastic behavior. Blends HC2 and HC4 could not form thermo-irreversible gel, which may be because of their relative lower CL concentration and the interruption of HPMC molecules. For these samples, the G’ was closer to G” with the increasing CL content. The LVR was ≤0.64 ± 0.15%, ≤1.17 ± 0.15%, ≤1.17 ± 0.15%, ≤0.44 ± 0.10%, and ≤0.37 ± 0.05% for HC0, HC2, HC4, HC6, and HC8, respectively.

Compared with blends at 25 °C, although the LVR of samples at 82 °C was narrower, its gel strength (G’ in the LVR range) at 82 °C was larger, for example, with HC8 at 82 °C, it showed G’ of about 60 times larger than the blend at 25 °C. The reason for the tremendous changes of strength at 82 °C may be that HPMC gel is thermo-reversible, and that CL can form stronger gel structure at higher temperatures, and that there might be an inter-network between these two thermal gels. Compared with HPMC, the G’ and G” of the HPMC/CL blends increased with the increasing content of CL at both temperatures. The increase extent of the blends at the high temperature was higher than that at the low temperature, which may be because both CL and HPMC formed gel networks at high temperatures and co-gel may have been formed.

### 3.2. The Effect of CL on the Extent of Gel-Like Behavior of the HPMC System

**Figure 2:**
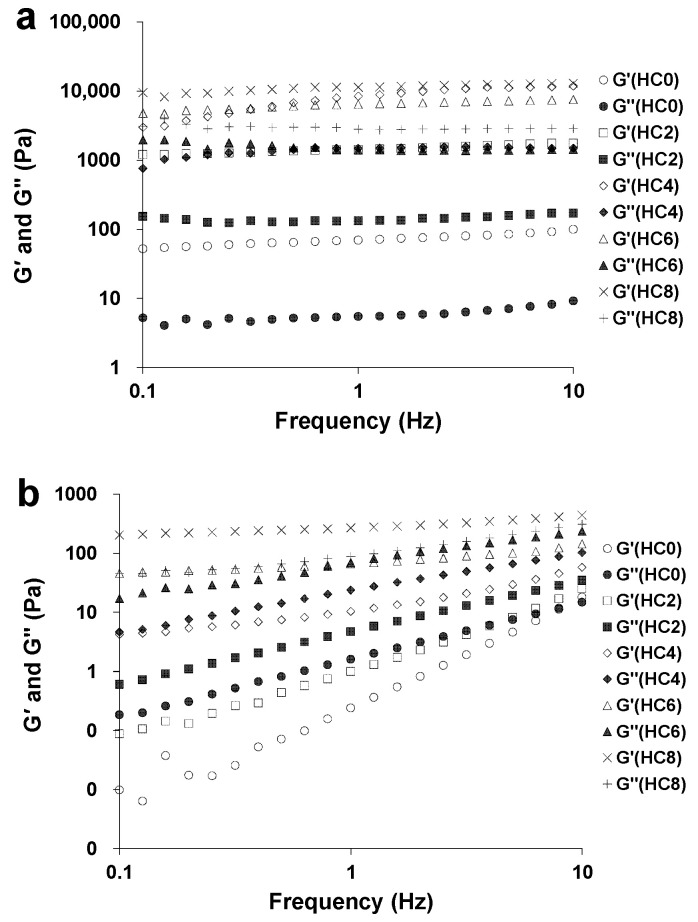
Frequency sweep curves of HPMC/CL blends at 82 °C (**a**) and 25 °C (**b**).

Figure 2a shows a frequency sweep curve of HPMC/CL blends at 82 °C. The G’ and G” of samples did not obviously change with the increased frequency, and G’ was always larger than G”, meaning that the samples exhibited gel-like behavior. Figure 2b displays the frequency sweep curve of HPMC/CL blends at 25 °C. G’ and G” increased with the increasing frequency. For HC0, HC2, and HC4, G” was larger than G’ in the entire frequency range, meaning that samples exhibited sol-like behavior. For HC6, at a lower frequency, the G’ was larger than G”, while at a higher frequency, G’ was smaller than G”, meaning that the gel structure was disrupted. For HC8, G’ was larger than G” at the tested frequency range, meaning that samples exhibited gel-like behavior.

The observed strong increase of both moduli with increasing CL content at both lower and higher temperatures in Figure 2 is normal, which could be explained by macromolecular entanglement phenomena. Since higher polymer concentration can increase the entanglement density (the number of intermolecular contacts per unit volume), the viscoelastic properties increase correspondingly [10]. At lower CL concentration, the polymer chains have more space to relax to a more favorable state by slippage of the entanglement point of chains. However, with increasing CL content, the available space for the polymer chains to relax declines gradually. The chains in the blending samples can no longer slip past one another readily, and the entanglement points act more like fixed network anchors. Consequently, the ability of this entanglement network to temporarily store the imposed energy increases, and the network of the blending samples behaves more like elastic solid, and the contribution of elasticity of the samples increase [24].

### 3.3. The Effect of CL on the Sol-Gel Transition of HPMC Solution

**Figure 3:**
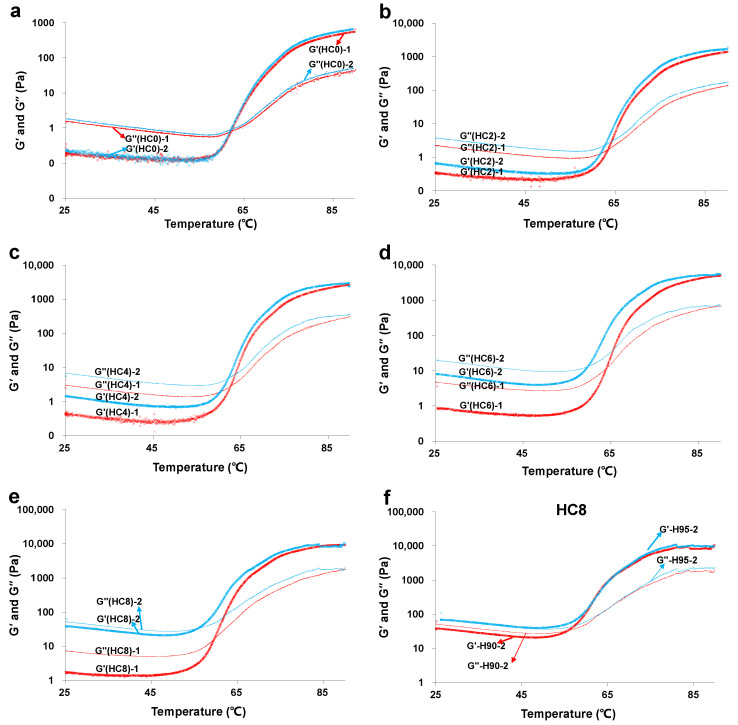
Temperature sweep curves for the first and second sweeps of the HPMC/CL blends: HC0 (**a**); HC2 (**b**); HC4 (**c**); HC6 (**d**); HC8 (**e**); and the second temperature sweeps of HC8 after the first temperature sweep heating at 90 °C and 95 °C, respectively (**f**).

A temperature sweep of HPMC/CL blends was carried out twice, and the obtained sweep curves are illustrated in Figure 3. The sweeps for the first time and second time are differentiated using different colors and are written as −1 and −2, respectively.

#### 3.3.1. The Effect of CL on the Sol-Gel Transition of HPMC Solution for the First Time

The effective molecular chain hardness, junction zone strength, and bond quantities contribute to G’ and friction energy consumption in a liquid state, which includes mobility, movement, friction of small molecules, and the vibration and rotation of functional groups all contribute to the value of G” [25,26]. These moduli can be used to analyze gelation behavior, speed of gel network formation, and structure characteristics [25]. They also reflect the inner structural developments and molecular interactions during gel network formation [27]. The G’–G” intersection indicates the sol-gel transition. The temperature sweep curves carried out for the first time are marked in red. Seen from the red curves in Figure 3a–e, all the samples exhibited three stages during the heating process: the initial platform zone, the gel structure formation zone, and the end platform zone. In the stage of the initial platform, the values of G’ and G” tended to decrease slightly with the increasing temperature, which may be because of the normal temperature thinning phenomena. When the temperature increased further, both G’ and G” rose rapidly and the intersection of G’ and G” appeared, which was the gel formation stage. HPMC can form gel structure due to hydrophobic interactions of methoxy groups and hydrogen interactions in molecules [28], and CL can form a single-helix through hydrogen bonds or a triple-stranded helix gel structure through hydrophobic interactions [17,18]. Other than these gel structures, co-gel structures may also form. The gel formation temperatures of the first temperature sweeps can be seen from Table 1. For HC0, the gel formation temperature was 63.0 °C, while the gel formation temperatures of HC2, HC4, HC6, and HC8 were 63.5 °C, 63.0 °C, 62.1 °C, and 60.2 °C, respectively. From the results of the significance analysis, for HC8, the gel formation temperature significantly decreased. This may be because a higher content of CL may promote the formation of CL gel and co-gels. In the end platform, the values of G’ and G” were high and stable, indicating that the gel network of the sample formed completely.

The G’ and G” values of the samples in all the three stages increased with the increasing CL content. At a higher temperature, the G’ and G” of all the samples increased tremendously with the increasing CL content. The increasing extent was especially tremendous for HC8. At lower temperatures (lower than the gel formation temperature), the G’ and G” values of samples HC0, HC2, HC4, HC6, and HC8 were small, and the increasing extents with increasing CL content were also small, corresponding to their liquid properties at a lower temperature.

#### 3.3.2. The Effect of CL on the Repeated Sol-Gel Transition of HPMC Solution

From Figure 3a–e, the two temperature sweep curves of HPMC/CL blends can be compared. The two temperature sweep curves of the pure HPMC sample were completely coincidental, indicating that HPMC is a completely thermo-reversible gel. Meanwhile, the two curves of samples containing CL showed some differences. For blending samples HC2, HC4, HC6, and HC8, the moduli (G’ and G”) of the second temperature curves were higher than that of the first sweep. The increase of moduli for the second sweep may be because, as a whole, blending samples do not show thermal-irreversible gel properties, while a little part of CL may keep thermal-irreversible properties after the first temperature sweep, keeping its elasticity and viscosity when re-scanning. The increasing of the moduli for the second sweep was more outstanding at a lower temperature and with increasing CL content.

As shown in Table 1, the gel formation temperatures were 63.0 °C, 63.5 °C, 63.0 °C, 62.1 °C, and 60.2 °C for HC0, HC2, HC4, HC6, and HC8, respectively, for the first sweep, and 63.3 °C, 62.9 °C, 62.3 °C, 60.5 °C, and 56.5 °C, respectively, for the second sweep. Based on the results of the significance analysis, for HC6 and HC8, the gel formation temperature of the second sweep was significantly lower than that of the first sweep. This may be because, as a whole, HC6 and HC8 do not show thermal-irreversible behavior, while a part CL in these samples still existed in a gel state when re-scanning, which brought the gel formation forward. For the second temperature sweep, both blends containing 6% and 8% CL showed a significant decrease of gel formation temperature, and the decreasing extent was much more obvious with the increasing CL content, which may also be because the higher content of CL may promote the formation of CL gel and co-gels.

One thing to mention: for the second temperature sweep, HC6 and HC8 did not show gel properties at 25 °C, while the strain sweep and frequency sweep of HC6 and HC8 in Figure 1b and Figure 2b show that HC6 and HC8 was in a gel state at 25 °C. This contradictory phenomenon was due to the different pretreating conditions. For the second temperature curve, the first temperature sweep, which was heated to 90 °C, could be regarded as its pretreating process. Meanwhile, for the strain sweep and frequency sweep, the pretreating process was heated to 95 °C and kept for 5 min. Therefore, the pretreating for the second temperature sweep was not enough to facilitate the formation of thermal-irreversible gels, resulting in HC6 and HC8 with liquid properties at a lower temperature when re-scanning. This is confirmed by Figure 3f, showing the second temperature sweeps of HC8, after the first temperature sweep heated to 90 °C and 95 °C. After heating to 95 °C, the sample at a lower temperature for the repeated sweep behaved like a gel property (G’ > G’’); after the pretreating to 90 °C, the sample at a lower temperature for the repeated temperature sweep still behaved as a liquid property (G’ < G’’). This proves that heating to 95 °C can result in the formation of thermal irreversible gel of HC8. Tada, Matsumoto, and Masuda [17] stated that CL can form thermal-irreversible gel after heating to 80 °C, but combing with our experimental results, this temperature may not be absolute or proper for CL-based blends or different concentrations of the CL system.

### 3.4. The Effect of CL on the Viscosity of the HPMC System

**Figure 4:**
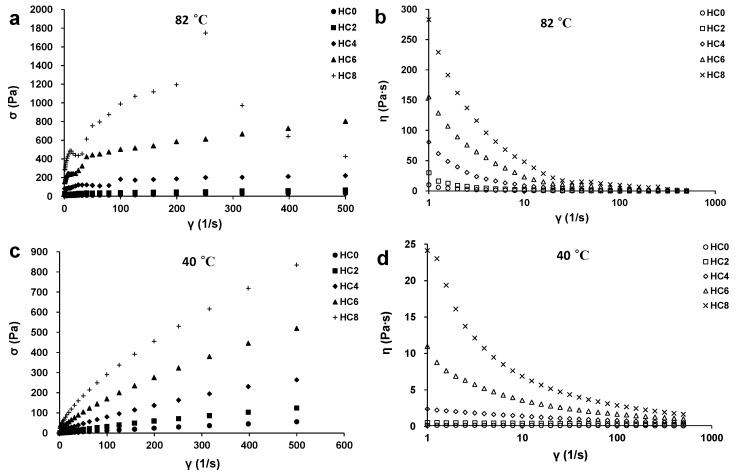
Shear stress and viscosity curves of HPMC/CL blends at 82 °C (**a**,**b**) and 40 °C (**c**,**d**).

Figure 4 shows the shear stress–shear rate sweep curve of HPMC/CL blends at 40 °C and 82 °C. For samples at the same temperature, the viscosity of the sample increased with the increasing CL content. The viscosity of all the samples decreased with the increasing shear rate, meaning that all the samples exhibited shear thinning behaviors. At a higher shear rate, the original arrangements of samples were disrupted, molecular chains of the sample were aligned along the direction of the shear stress, and the shear resistance was reduced, resulting in a decrease of the viscosity.

According to fluid Equations (1) or (2) in Section 2.3, the shear stress–shear rate results were fitted, and the *n*, *K*, σ_y_, and r^2^ values of the HPMC with different CL contents at different temperatures are calculated and displayed in Table 2. At 82 °C, HC8 did not conform to these monotonous functions, since a negative slope appeared; a negative slope in steady flow curves is an indication of instabilities, which are due to shear banding, wall slip, etc. [29]. Equation (2) can be used to fit the results of all the other samples at 82 °C, and the fitting results were relatively good based on the value of r^2^, meaning that they were all yield-shear thinning fluids, which are also recognized as Herschel-Bulkley fluids. For yield-shear thinning fluids, due to the attraction between intermolecular forces, hydrogen bonds, and hydrophobic forces, three-dimensional gel network structures can be formed at rest. These network structures are just like solids under low stress, which only produce elastic deformation and do not flow. Only when the external force exceeds a certain critical value, the gel network structure will be destroyed and the fluid will flow. The *σ_y_* value increased with the increasing CL content, with HC4 and HC6 showing significant increases, meaning that these two blends showed more obvious solid-like behavior. Meanwhile, Equation (1) can be used to fit the results of all the samples at 40 °C, and the fitting results are very well based on the value of r^2^, meaning that the samples at 40 °C were all shear thinning fluids. The *n* value of Newtonian fluid is 1, while, for shear thinning fluids, the *n* value is less than 1. The more the value deviates from 1, the clearer the shear thinning behavior [12]. For samples at the same temperature, with the increase of CL content, the *n* value decreased and the *K* value increased, meaning that samples with more CL showed stronger shear thinning behavior and increased viscosity. With the increase of CL, the concentration of polymer increased, which increased the molecular chain density per unit volume and the probability of hydrogen and hydrophobic interactions between the molecules, resulting in a more complex arrangement of the samples. Meanwhile, after shearing, the interactions of molecules were destroyed, with the molecule chains being arranged simply and linearly along the direction of the shear stress. Samples with higher CL content were subjected to more disruption of molecular interactions after shearing, thus a stronger shearing thinning extent and larger decreasing extent of viscosity were observed for these samples.

For the samples with the same CL content, the viscosity of HPMC/CL blends were in the order: 82 °C > 40 °C. A much higher viscosity at a higher temperature was due to the gel structure formation. The shear thinning extent of samples also increased with the increasing temperature because gels at higher temperatures underwent much more disruption of molecular interactions than the solution at lower temperatures, since gels are compact network structures. The blends had higher gel strength and viscosity at higher temperatures and were anticipated to be used as thickening agents for hot drinks, skeletons for bakery products, and pre-coatings for fried foods as water retention scherm.

### 3.5. Morphology of HPMC/CL Blends

**Figure 5:**
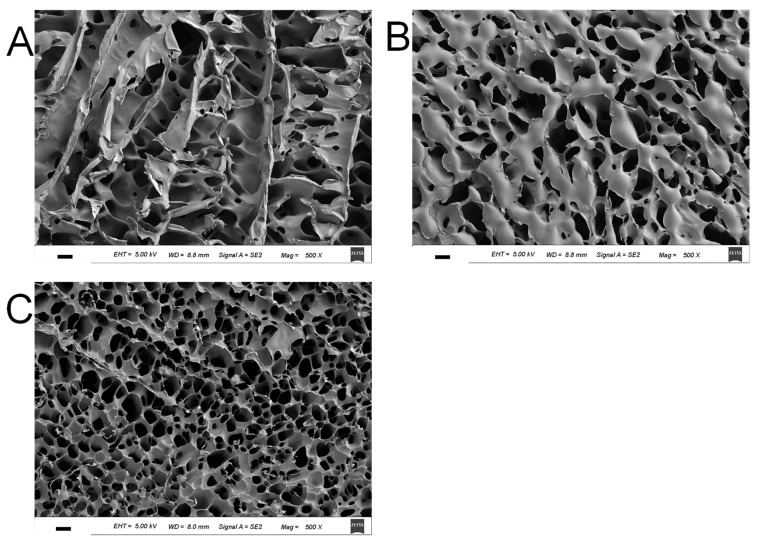
SEM images for the samples HC0 (**A**), HC2 (**B**), and HC6 (**C**). The scale bar is 10 μm.

The morphological structures of HPMC/CL blends incubated at 82 °C are shown in Figure 5. The surface of the pure HPMC gel exhibited a porous structure, with elongated or round holes. The hole sizes of pure HPMC were relatively large. After the addition of 2% CL, blending gels had many more holes, which were smaller. After the addition of 6% CL, the blending gel had a regular round, smaller, and denser porous structure, indicating the formation of much more compact gels. To conclude, with the increasing CL content, the pore sizes decreased, which contributed to the larger G’, the gel strength, and the viscosity of blending gels.

## 4. Conclusions

The establishment of this thermal–thermal HPMC/CL blending gel system and the research on its rheological properties provide some methodological and theoretical references for other thermal–thermal blending gels. The viscosity, moduli, gel strength, and compactness of the gel network of HPMC/CL blends at 82 °C were tremendously higher than that at a lower temperature, which provides some theoretical guidance for their application, such as, thickening agents for hot drinks, skeletons for bakery products, and pre-coatings for fried foods as water retention scherm.

## Figures and Tables

**Table 1:** Gel formation temperatures of the first (T_1_) and second (T_2_) temperature sweeps of HPMC/CL blends.

	HC0	HC2	HC4	HC6	HC8
T_1_ (°C)	63.0 ± 1.2 ^a^	63.5 ± 0.2 ^a^	63.0 ± 0.5 ^a^	62.1 ± 0.9 ^a^	60.2 ± 1.0 ^b^
T_2_ (°C)	63.3 ± 1.1 ^a^	62.9 ± 0.3 ^a^	62.3 ± 0.3 ^a^	60.5 ± 1.0 ^b^	56.5 ± 0.5 ^c^

Different superscript letters mean that values differ significantly (*p* < 0.05).

**Table 2:** Flow behavior index (*n*), fluid consistency index (*K*), yield stress (*σ_y_*), and regression coefficient (r^2^) of HPMC/CL blends at 82 °C and 40 °C, respectively.

		*n*	*K*	*σ_y_*	r^2^
82 °C	HC0	0.65 ± 0.14 ^a^	0.41 ± 0.051 ^b^	6.9 ± 6.6 ^b^	0.9819 ± 0.0094
	HC2	0.50 ± 0.15 ^ab^	3.0 ± 2.9 ^b^	16 ± 4.0 ^b^	0.9240 ± 0.0527
	HC4	0.38 ± 0.073 ^b^	18 ± 9.7 ^b^	53 ± 23 ^a^	0.8707 ± 0.0493
	HC6	0.36 ± 0.056 ^b^	86 ± 44 ^a^	67 ± 15 ^a^	0.9625 ± 0.0358
	HC8	--	--	--	--
40 °C	HC0	0.95 ± 0.010 ^a^	0.16 ± 0.017 ^e^	--	0.9998 ± 0.0001
	HC2	0.89 ± 0.053 ^b^	0.56 ± 0.18 ^d^	--	0.9991 ± 0.0005
	HC4	0.76 ± 0.027 ^c^	2.4 ± 0.13 ^c^	--	0.9997 ± 0.0002
	HC6	0.65 ± 0.0080 ^d^	8.7 ± 0.20 ^b^	--	0.9971 ± 0.0003
	HC8	0.58 ± 0.0030 ^e^	20 ± 0.32 ^a^	--	0.9915 ± 0.0033

The different parameter values (*n*, *K*, and *σ_y_*) at each temperature were compared separately. Different superscript letters mean that values differed significantly (*p* < 0.05).

## Data Availability

The data presented in this study are available on request from the corresponding author. The data are not publicly available due to technical or time limitations.
